# Continuous gas/liquid–liquid/liquid flow synthesis of 4-fluoropyrazole derivatives by selective direct fluorination

**DOI:** 10.3762/bjoc.7.120

**Published:** 2011-08-02

**Authors:** Jessica R Breen, Graham Sandford, Dmitrii S Yufit, Judith A K Howard, Jonathan Fray, Bhairavi Patel

**Affiliations:** 1 Department of Chemistry, Durham University, South Road, Durham, DH1 3LE, UK; 2 Chemical Crystallography Group, Department of Chemistry, Durham University, South Road, Durham, DH1 3LE, UK; 3 Pfizer Global Research & Development, Ramsgate Road, Sandwich, Kent, CT13 9NJ, UK

**Keywords:** continuous flow reactions, fluorine, fluoropyrazole, gas-liquid flow reactor, selective direct fluorination

## Abstract

4-Fluoropyrazole systems may be prepared by a single, sequential telescoped two-step continuous gas/liquid–liquid/liquid flow process from diketone, fluorine gas and hydrazine starting materials.

## Introduction

Organic systems which bear fluorine atoms are used in an ever widening range of applications in the life sciences. Many commercially significant pharmaceutical and agrochemical products [1–3] owe their biological activity to the presence of fluorinated groups within their structure. Since carbon–fluorine bonds are rare in naturally occurring organic molecules [4], an efficient, selective and economically viable methodology for the synthesis of fluoroorganic derivatives is required to exploit fully the use of fluorinated systems in life science applications. In general, there are two complimentary approaches to the synthesis of fluoroorganic products [5–6], which involve either carbon–fluorine bond formation, requiring functional group interconversion utilising an appropriate nucleophilic or electrophilic fluorinating agent [6], or syntheses based on the reactions of appropriate fluorine containing building blocks [7]. Of course, whichever methodology is used for the synthesis of a specific fluorinated organic molecule, a carbon–fluorine bond must be formed at some stage of the synthetic process, and various fluorinating agents have been developed over many years in attempts to meet synthetic requirements, with varying degrees of success [5].

In an on-going research programme at Durham, aimed at developing widely applicable an effective, selective, direct fluorination methodology, we have been exploring the use of elemental fluorine, a previously underused reagent in organic chemistry, for the synthesis of fluoroorganic systems [8–10]. Methodologies for the preparation of, for example, a range of fluorinated aliphatic [11], carbonyl [12–14], aromatic [15] and heterocyclic [16–17] systems have been established and scaled-up by our industrial collaborators for use in the synthesis of commercially important pharmaceutical intermediates [18]. As part of our studies, aimed at further control of the direct fluorination procedures for larger scale manufacturing, we developed continuous flow microreactor systems that enabled gas/liquid fluorination reactions between fluorine and various substrates to occur in very efficient processes [19–21].

Fluoro-carbonyl derivatives can, in principle, be utilised as building blocks for the preparation of more complex systems such as fluorinated terpenoids, steroids and a range of heterocyclic systems, upon appropriate synthetic elaboration [7], and, consequently, there is much interest in the development of a synthetic methodology for the preparation of such useful fluorinated intermediates. It has been established that, in both batch and continuous flow processes, 1,3-dicarbonyl derivatives are not equally reactive towards fluorine gas [12] and that the ease of selective direct fluorination depends on the nature and proximity of other functional groups. Substrates that have a high initial equilibrium enol concentration in the reaction media that are used for fluorination reactions, such as formic acid or acetonitrile, and/or rapidly convert from the keto to the enol form, will react rapidly and selectively with fluorine to give monofluorinated products in high yield. Conversely, substrates that have low enol concentrations at equilibrium and/or slow keto-enol exchange rates give low yields of the desired monofluorinated dicarbonyl products [12]. Indeed, for carbonyl systems with low enol equilibrium contents and/or low keto-enol exchange rates, direct fluorination must be carried out in conditions that enhance enol formation by base catalysis, by metal catalysis or by fluorination of appropriate pre-formed enol derivatives, such as trimethylsilyl enols or enol acetate derivatives [13–14].

Dicarbonyl systems are, of course, widely used for the construction of heterocyclic ring systems such a pyrimidine, pyridazine and pyrazole derivatives [22–23]. Of relevance to this paper, pyrazole and its derivatives constitute an important class of compounds, which exhibit various biological and pharmaceutical activities ranging from antitumor to anti-inflammatory, antipsychotic, antimicrobial, antiviral and antifungal activities. Pyrazoles are also useful intermediates for many industrial products and it is, therefore, not surprising that many synthetic methods have been developed for the preparation of such heterocyclic systems, for example, through 1,3-dipolar cyclo-additions of diazo compounds and the direct condensation of 1,3-diketones and hydrazines [23].

However, the synthetic methodology for the preparation of the corresponding fluoropyrazole derivatives has not been developed to any great extent despite the potential use of such systems in life science applications. Recently, we explored direct fluorination of various pyrazole substrates using elemental fluorine and, in many cases, obtained low yields of the desired fluoropyrazole products due to significant tar formation [24], while the corresponding fluorination of a limited number of pyrazole systems by Selectfluor^TM^ has been described by other researchers [25]. Various building block strategies that yield fluoropyrazole derivatives from, for example, reactions of appropriate fluorodicarbonyl [26] and fluorocyano-ketones [27], have also been reported.

Consequently, we aimed to develop an effective, selective continuous flow methodology for the efficient synthesis of fluoropyrazole systems and, in this paper, we demonstrate that fluorination of diketones to corresponding fluorodiketones, followed by sequential cyclization to the appropriate fluoropyrazoles upon reaction with a hydrazine derivative, can be accomplished in a single, two-step, telescoped, gas/liquid–liquid/liquid continuous flow process. No examples of gas/liquid–liquid/liquid processes involving direct fluorination as the first stage of a continuous flow procedure have been reported previously.

## Results and Discussion

**Figure 1:**
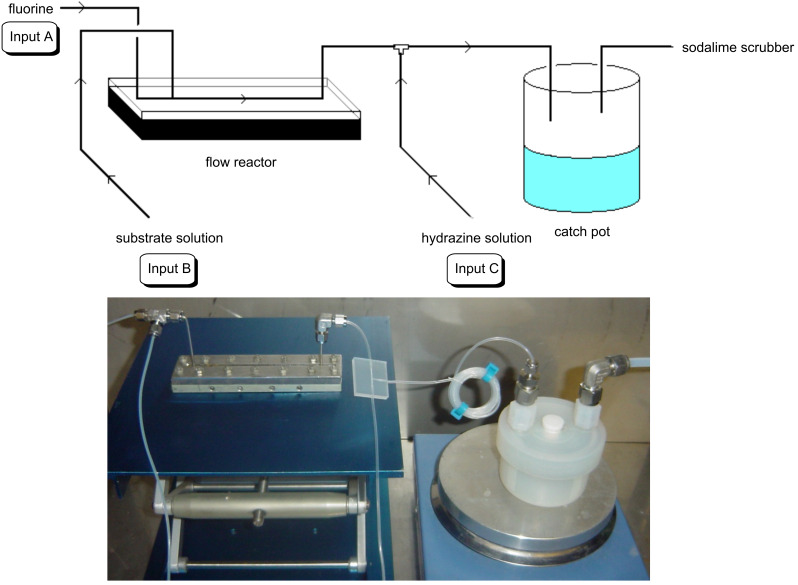
Sequential gas/liquid–liquid/liquid flow reactor for the synthesis of 4-fluoropyrazole derivatives.

After some development work, a continuous flow reactor for sequential gas/liquid–liquid/liquid synthesis of fluoropyrazoles from fluorine, hydrazine and diketone starting materials was constructed from nickel metal and narrow bore PTFE tubing as described previously [28–29] (Figure 1).

Briefly, fluorine gas, diluted to 10% v/v solution in nitrogen was added via a mass flow controller to the microchannel through Input A, the diketone substrate dissolved in acetonitrile was added by a syringe pump into the microchannel via Input B and was made to react with fluorine while both these starting materials passed down the reactor channel in a ‘pipe flow’ regime, as observed in previous direct fluorination reactions using this reactor design. The hydrazine solution was added by a syringe pump, via a T-piece at Input C, into the liquid flow, which was carried along the flow reaction channel by the pressure of the accompanying nitrogen gas. The crude reaction mixture was then passed into a vessel containing water to quench the reaction and neutralise any excess HF. Work-up by extraction of the crude reaction mixture with dichloromethane, drying and evaporation of the organic solvent gave a crude product which was further purified by column chromatography or recrystallisation if required.

In our initial exploratory reactions, pentane-2,4-dione (**1a**) was used as the substrate, because this system has a high initial enol content at equilibrium and rapidly enolises in acetonitrile solution [12]. By varying the flow rate of fluorine gas we were able to achieve reaction conditions that would convert all of the diketone substrate to a fluorodiketone in the first stage of the continuous process. Subsequent separation of fluoropyrazole (**4a**) from the corresponding non-fluorinated pyrazole, formed by the coupling of not reacted pentane-2,4-dione and hydrazine, was difficult to achieve. Any remaining difluorinated diketone formed by excess fluorination of the substrate was not involved in the second cyclization process and remained in aqueous solution in the collection vessel.

In initial experiments, 1 mmol of the pentane-2,4-dione (**1a**) was diluted in 4 mL of acetonitrile, and 1.2 mmol of hydrazine hydrate (**3a**) was also dissolved in 4 mL of solvent (either acetonitrile, ethanol or water). Both were added concurrently to the flow reactor at the rate of 2 mL/min into Inputs B and C, respectively, using accurate syringe pumps. Excess 10% F_2_/N_2_ gas mixture was passed into the flow channel at a rate of 18 mL/min to achieve full conversion of pentane-2,4-dione to the corresponding 3-fluoropentane-2,4-dione, and subsequent formation of the fluoropyrazole product **4a** occurred with no non-fluorinated pyrazole by-product observed. Early investigations showed that some of the 3-fluoropentane-2,4-dione remained unchanged when only 1.2 mmol of the hydrazine was used and, therefore, in subsequent reactions excess hydrazine (1.5 mmol) was added to ensure complete conversion of the 3-fluoropentane-2,4-dione to the 4-fluoropyrazole **4a** (Table 1).

**Table 1:** Synthesis of 4-fluoro-3,5-dimethylpyrazole derivatives.

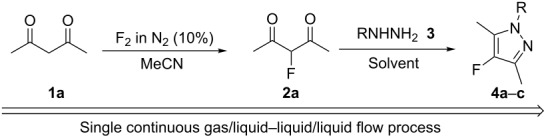			

Diketone **1**	Hydrazine **3**	Solvent	4-Fluoropyrazole **4** Yield

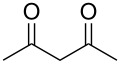			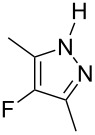
**1a**	NH_2_–NH_2_·H_2_O, **3a**	H_2_O	**4a**, 74%
**1a**	**3a**	EtOH	**4a**, 66%
**1a**	**3a**	MeCN	**4a**, 77%
			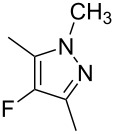
**1a**	MeNH–NH_2_, **3b**	H_2_O	**4b**, 83%
**1a**	**3b**	EtOH	**4b**, 73%
**1a**	**3b**	MeCN	**4b**, 68%
			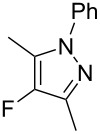
**1a**	PhNH–NH_2_, **3c**	EtOH	**4c**, 72%
**1a**	**3c**	MeCN	**4c**, 67%

In all reactions, acetonitrile was used as the solvent for the fluorination stage as this reaction medium has been found to be very effective for direct fluorination reactions of dicarbonyl systems [12]. The hydrazine was added to the continuous flow process dissolved in either acetonitrile, water or ethanol depending on the solubility of the hydrazine derivative. Water and ethanol are miscible with acetonitrile, thus enabling the cyclisation process to occur by efficient mixing of the two flow streams within the reactor channel.

Similarly, fluoropyrazole derivatives **4b** and **4c** were prepared by reaction of **1a** with fluorine and methyl hydrazine **3b** and phenyl hydrazine **3c**, respectively, and these results are included in Table 1.

With the conditions for gas/liquid–liquid/liquid processes established from reactions involving pentane-2,4-dione (**1a**), several other fluoropyrazole systems **4d**–**h** were synthesised from a series of related diketone starting materials **1b**–**f** in one continuous flow process from hydrazine **3a** and these are collated in Table 2.

**Table 2:** Synthesis of 4-fluoropyrazole derivatives.

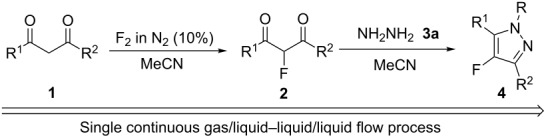	

Diketone **1**	4-Fluoropyrazole **4** Yield

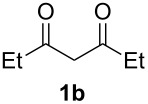	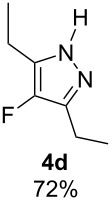
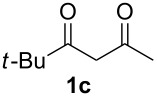	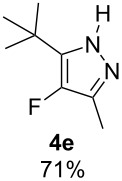
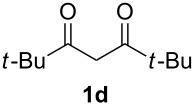	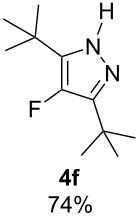
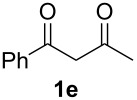	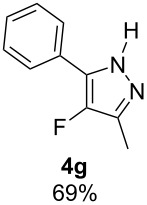
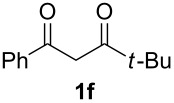	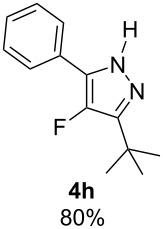

All fluoropyrazole products **4a**–**h** were isolated and purified, and then characterised by NMR spectroscopy and mass spectrometry techniques; results were compared to literature data where available. ^19^F NMR spectra of all 4-fluoropyrazole products show singlets at ~ −175 to −185 ppm, consistent with literature values.

**Figure 2:**
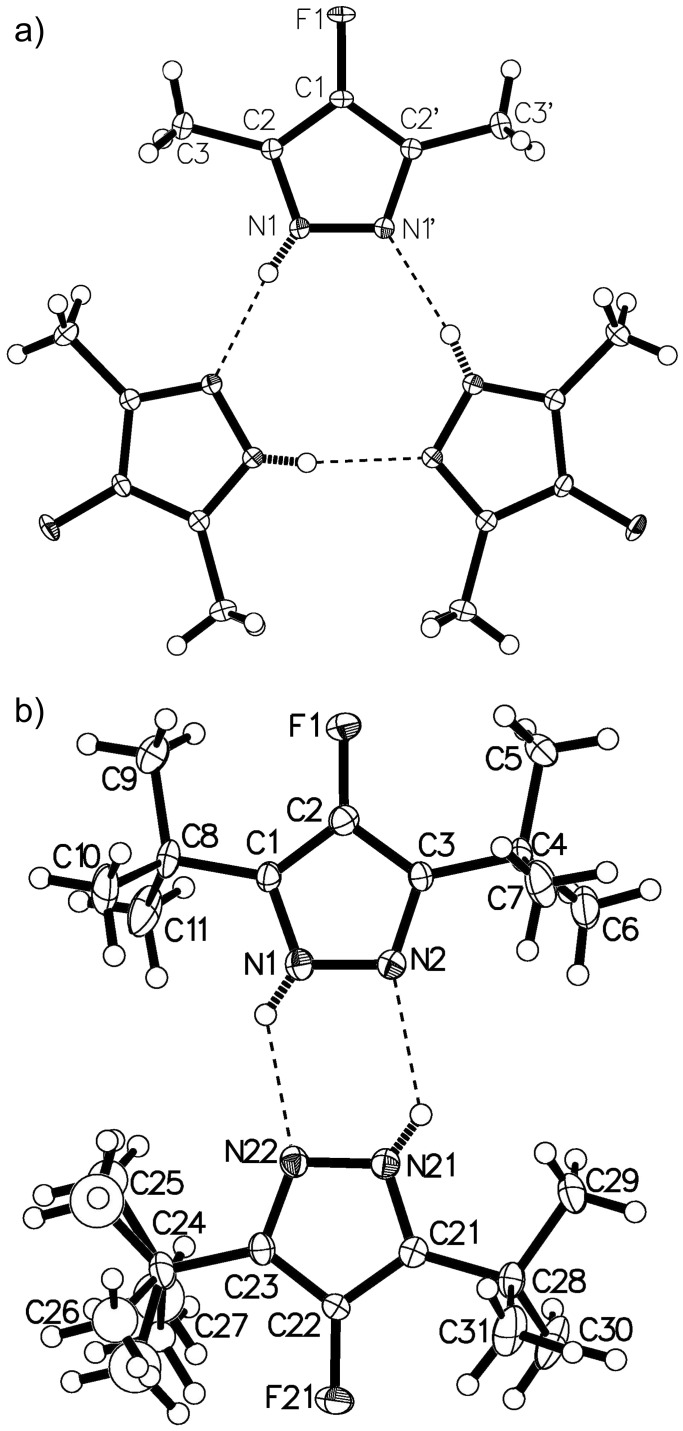
H-bonded cycles in structures **4a** (a) and **4f** (b) (only one disordered pyrazole hydrogen atom is shown in each case).

Furthermore, the structures of **4a** and **4f** were confirmed by X-ray crystallography (Figure 2). In both cases, the 5-membered rings are planar and the pyrazole hydrogen atoms are disordered over two positions. The molecules of both compounds in the crystals are linked together in H-bonded cycles. In the case of **4a**, the cycles are R^3^_3_(9) trimers [30], while the presence of bulky *t*-Bu groups in **4f** results in the formation of R^2^_2_(6) dimers (Figure 2).

## Conclusion

4-Fluoropyrazole derivatives were synthesised by sequential direct fluorination of appropriate 1,3-diketones and subsequent cyclisation of the in situ generated fluorodiketone with a hydrazine derivative. This represents the first example of a sequential, continuous flow gas/liquid–liquid/liquid process involving direct fluorination in the first stage of a multi-step, telescoped, continuous flow process. Thus, continuous flow methodology was used for successful sequential carbon-fluorine bond formation and subsequent fluorine-containing building block reactions in a single continuous high yielding, efficient and regioselective process.

## Experimental

Synthetic procedures for the preparation of all the 4-fluoropyrazole compounds described in this paper are given either below or in Supporting Information File 1. Supporting Information File 2 contains copies of the NMR spectra.

### Two step process – general procedure

After purging the continuous flow reactor apparatus [29] (Figure 1) with nitrogen, a 10% mixture of fluorine in nitrogen (v:v) was passed through the flow reactor via Input A at a prescribed flow rate that was controlled by a gas mass flow controller (Brooks Instruments). The flow reactor was cooled by an external cryostat to 5–10 °C. The diketone solution was injected by a mechanised syringe pump into the flow reactor channel at a prescribed flow rate through the substrate Input B. At the same time, the hydrazine mixture was injected by a mechanised syringe pump at Input C, into the flow reactor via a T-piece, at a prescribed flow rate. All flow streams were passed through the reactor and the product mixture was collected in a vessel containing water. The collected mixture was then extracted from DCM (3 × 30 mL) and washed with sodium bicarbonate (30 mL) and water (30 mL). The combined extracts were then dried (MgSO_4_), filtered and the solvent evaporated to give a solid residue, which was purified by recrystallisation or column chromatography on silica gel to give the desired 4-fluoropyrazole product.

#### 4-Fluoro-3,5-dimethyl-1*H*-pyrazole (4a)

Pentane-2,4-dione (**1a**) (0.10 g, 1.0 mmol) in MeCN (4 mL, 2 mL/h), fluorine (8 mL/min), and hydrazine hydrate (**3a**, 0.07 g, 1.5 mmol) in ethanol (4 mL, 2 mL/h), after purification by column chromatography on silica gel with hexane and ethyl acetate (1:1) as the eluent, gave 4-fluoro-3,5-dimethyl-1*H*-pyrazole (**4a**) (0.075 g, 66%) as pale yellow crystals; mp 107–109 °C (lit. [31]: mp 108–110 °C); ^1^H NMR (400 MHz, CDCl_3_) δ 2.26 (s, 6H, CH_3_), 8.61 (br s, 1H, NH); ^13^C NMR (126 MHz, CDCl_3_) δ 9.1 (d, ^3^*J*_CF_ 2.9 Hz, CH_3_), 129.6 (br s, C-3), 143.5 (d, ^1^*J*_CF_ 239.8 Hz, C-4); ^19^F NMR (376 MHz, CDCl_3_) δ −183.4 (s); MS (EI^+^) *m*/*z:* 114.1 ([M]^+^, 100%), 113.0 (82), 41.9 (79); HRMS (*m*/*z*): [M + H]^+^ calcd for C_5_H_7_FN_2_, 115.0672; found, 115.0667.

## Supporting Information

File 1 Experimental data.

File 2 NMR spectra.
