# Supplementary material for: Continuous gas/liquid–liquid/liquid flow synthesis of 4-fluoropyrazole derivatives by selective direct fluorination
Source: Beilstein J Org Chem. 2011 Aug 2;7:1048–54. doi: 10.3762/bjoc.7.120 (PMC3168861; doi:10.3762/bjoc.7.120)
Supplement: File 1 — Experimental data. [file Beilstein_J_Org_Chem-07-1048-s001.pdf]

Supporting Information File 1

for

Continuous gas/liquid–liquid/liquid flow synthesis of 4-fluoropyrazole derivatives by selective direct fluorination

Jessica R. Breen[1], Graham Sandford[1*], Dmitrii S. Yufit[2], Judith A. K. Howard[2], Jonathan Fray[3] and Bhairavi Patel[3]

Address: [1]Department of Chemistry, Durham University, South Road, Durham, DH1 3LE, UK; [2]Chemical Crystallography Group, Department of Chemistry, Durham University, South Road, Durham, DH1 3LE, UK and [3]Pfizer Global Research & Development, Ramsgate Road, Sandwich, Kent, CT13 9NJ, UK

Email: Graham Sandford* - Graham.Sandford@durham.ac.uk

*Corresponding author

Experimental data

4-Fluoro-1,3,5-trimethyl-1*H*-pyrazole (4b)

Pentane-2,4-dione (**1a**) (0.10 g, 1.0 mmol) in MeCN (4 mL, 2 mL/h), fluorine (18 mL/min), and methyl hydrazine (**3b**) (0.07 g, 1.5 mmol) in ethanol (4 mL, 2 mL/h), after recrystallization of the crude product from hexane gave 4-fluoro-1,3,5-trimethyl-1*H*-pyrazole (**4b**) (0.094 g, 73%) as yellow crystals; mp 83–85 °C; (Found: $[M-H]^+$, 127.0866. $C_6H_9FN_2$ requires: $[M-H]^+$, 127.0871); ^{1}H NMR (700 MHz, $CDCl_3$) δ 1.53 (3H, s, CH_3), 1.78 (3H, s, CH_3), 3.26 (3H, s, NCH_3); ^{13}C NMR (176 MHz, $CDCl_3$) δ 7.94 (d, $^3J_{CF}$ = 3.1 Hz, CH_3), 9.77 (d, $^3J_{CF}$ = 3.0 Hz, CH_3), 36.4 (s, NCH_3), 123.7 (d, $^2J_{CF}$ = 25.8 Hz, C-3), 133.0 (d, $^2J_{CF}$ = 10.9 Hz, C-5), 145.4 (d, $^1J_{CF}$ = 241.1 Hz, C-4); ^{19}F NMR (658 MHz, $CDCl_3$) δ −181.4 (s); *m/z* (ES^+) 128.8 ($[MH]^+$, 100%).

4-Fluoro-3,5-dimethyl-1-phenyl-1*H*-pyrazole (4c)

Pentane-2,4-dione (**1a**) (0.10 g, 1.0 mmol) in MeCN (4 mL, 2 mL/h), fluorine (18 mL/min) and phenyl hydrazine (**3c**) (0.162 g, 1.5 mmol) in ethanol (4 mL, 2 mL/h), after purification by column chromatography on silica gel with 1:1 hexane and ethyl acetate as the eluent, gave 4-fluoro-3,5-dimethyl-1-phenyl-1*H*-pyrazole (**4c**) (0.137 g, 72%) as a yellow oil; (Found: $[M]^+$, 190.0906. $C_{11}H_{11}FN_2$ requires: $[M]^+$, 190.0906); ^{1}H NMR (400 MHz, $CDCl_3$) δ 2.35 (3H, s, CH_3), 2.37 (3H, s, CH_3), 7.20–7.50 (5H, m, Ar-H); ^{13}C NMR (126 MHz, $CDCl_3$) δ 9.7 (d, $^3J_{CF}$ = 3.1 Hz, CH_3), 10.3 (d, $^3J_{CF}$ = 3.0 Hz, CH_3), 124.4 (C-3′), 127.6 (C-4′), 129.4 (C-2′), 135.9 (d, $^2J_{CF}$ = 11.2 Hz, C-3), 140.1 (C-1′), 146.8 (d, $^1J_{CF}$ = 243.4 Hz, C-4); ^{19}F NMR (376 MHz, $CDCl_3$) δ -179.9 (s); *m/z* (EI^+) 190.2 ($[M]^+$, 100%) 148.0 (24), 118.1 (32), 77.0 (50).

3,5-Diethyl-4-fluoro-1*H*-pyrazole (4d)

Heptane-3,5-dione (**1b**) (0.13 g, 1.0 mmol) in MeCN (4 mL, 2 mL/h), fluorine (18 mL/min) and hydrazine monohydrate (**3a**) (0.075 g, 1.5 mmol) in MeCN (4 mL, 2 mL/h), after column

chromatography over silica gel with ethyl acetate as the eluent, gave 3,5-diethyl-4-fluoro-1*H*-pyrazole **4d** (0.102 g, 72 %) as pale yellow crystals; (Found: $[M]^+$, 142.0893. $C_7H_{11}FN_2$ requires: $[MH]^+$, 142.0906); ^{1}H NMR (400 MHz, $CDCl_3$) δ 1.21 (6H, t, $^3J_{HF}$ = 7.7 Hz, CH_3), 2.58 (4H, q, $^3J_{HF}$ = 7.6 Hz, CH_2), 11.5 (1H, bs, NH); ^{13}C NMR (126 MHz, $CDCl_3$) δ 12.7 (s, CH_3), 17.5 (s, CH_2), 135.0 (br s, C-3), 144.6 (d, $^1J_{CF}$ = 239.6 Hz, C-4); ^{19}F NMR (376 MHz, $CDCl_3$) δ -184.7 (s); *m/z* (EI^+) 142.6 ($[M]^+$, 100%), 127.0 (90), 113.1 (40), 83.1 (18) 59.1 (22).

5-*tert*-Butyl-4-fluoro-3-methyl-1*H*-pyrazole (4e)

5,5-Dimethylhexane-2,4-dione (**1e**) (0.16 g, 1.0 mmol) in MeCN (4 mL, 2 mL/h), fluorine (18 mL/min) and hydrazine monohydrate (**3a**) (0.075 g, 1.5 mmol) in MeCN (4 mL), after column chromatography over silica gel with 1:1 hexane and ethyl acetate as the eluent, gave 3-tert-butyl-4-fluoro-5-methyl-1*H*-pyrazole (**4e**) (0.11 g, 71%) as yellow crystals; mp 129–131 °C; (Found: $[M]^+$, 157.1131. $C_8H_{13}FN_2$ requires: $[M]^+$,157.1141); ^{1}H NMR (400 MHz, $CDCl_3$) δ 1.34 (9H, s, $C(CH_3)_3$), 2.21 (3H, s, CH_3); ^{13}C NMR (126 MHz, $CDCl_3$) δ 9.11 (s, CH_3), 29.2 (s, $C(\underline{C}H_3)_3$), 31.6 (s, $\underline{C}(CH_3)_3$), 131.5 (d, $^2J_{CF}$ = 19.4 Hz, C-5), 140.9 (d, $^2J_{CF}$ = 15.5 Hz, C-3), 144.4 (d, $^1J_{CF}$ = 241.3 Hz, C-4); ^{19}F NMR (376 MHz, $CDCl_3$) δ -179.9 (s); *m/z* (EI^+) 156.1 ($[M - H]^+$, 100%), 141.1 (90), 113.1 (21), 101.1 (23).

3,5-Di-*tert*-butyl-4-fluoro-1*H*-pyrazole (4f)

2,2,6,6-Tetramethylheptane-3,5-dione (**1d**) (0.184 g, 1.0 mmol) in MeCN (4 mL, 2 mL/h), fluorine (18 mL/min) and hydrazine monohydrate (**3a**) (0.075 g, 1.5 mmol) in MeCN (4 mL, 2 mL/h) after recrystallization of the crude product from hexane gave 3,5-di-tert-butyl-4-fluoro-1*H*-pyrazole (**4f**) (0.148 g, 74 %) as yellow crystals; mp 176–178 °C; (Found $[MH]^+$, 199.1605 $C_{11}H_{19}FN_2$ requires: $[MH]^+$, 199.1605); ^{1}H NMR (700 MHz, $CDCl_3$) δ 1.33 (s,

CH_3); ^{13}C NMR (176 MHz, $CDCl_3$) δ 28.9 (d, $^4J_{CF}$ = 1.8 Hz, C($\underline{C}H_3)_3$), 31.4 (d, $^3J_{CF}$ = 3.4 Hz, $\underline{C}(CH_3)_3$), 142.1 (bm, C-3) 143.6 (d, $^1J_{CF}$ = 243.1 Hz, C-4); ^{19}F NMR (658 MHz, $CDCl_3$) δ -174.5 (s); *m/z* (EI^+) 198.2 ($[M]^+$, 100%), 183.2 (66), 127.1 (22), 57.2 (42).

4-Fluoro-3-methyl-5-phenyl-1*H*-pyrazole (4g)

1-Phenylbutane-1,3-dione (**1e**) (0.162g, 1.0 mmol) in MeCN (4 mL, 2 mL/h), fluorine (18 mL/min) and hydrazine monohydrate (**3a**) (0.075 g, 1.5 mmol) in MeCN (4 mL, 2 mL/h), after column chromatography on silica gel with 7:3 hexane and ethyl acetate as the eluent, gave 4-fluoro-5-methyl-3-phenyl-1*H*-pyrazole (**4g**) (0.121 g, 69%) as white crystals; mp 136–138 °C; (Found: $[MH]^+$,177.0831. $C_{10}H_9FN_2$ requires: $[MH]^+$, 177.0750); ^{1}H NMR (400 MHz, $CDCl_3$) δ 2.04 (3H, s, CH_3), 7.31–7.76 (5H, m, Ar-H), 7.79 (1H, bs, NH); ^{13}C NMR (126 MHz, $CDCl_3$) δ 8.7 (CH_3), 125.6 (d, $^2J_{CF}$ = 3.8 Hz, C-5), 128.1 (Ar), 128.8 (Ar), 129.2 (C-3), 145.1 (d, $^1J_{CF}$ = 246.8 Hz, C-4); ^{19}F NMR (376 MHz, $CDCl_3$) δ −179.9 (s); *m/z* (EI^+) 176.1 ($[M]^+$, 100%), 145.9 (22), 108.1 (17), 77.0 (35).

3-*tert*-Butyl-4-fluoro-5-phenyl-1*H*-pyrazole (4h)

5,5-Dimethyl-1-phenylpentane-2,4-dione (**1f**) (0.20 g, 1.0 mmol) in MeCN (4 mL, 2 mL/h) and hydrazine monohydrate (**3a**) (0.075 g, 1.5 mmol) in MeCN (4 mL, 2 mL/h), after column chromatography on silica gel with 7:3 hexane and ethyl acetate as the eluent, gave 3-tert-butyl-4-fluoro-5-phenyl-1*H*-pyrazole (**4h**) (0.174 g, 80%) as a yellow oil; (Found: $[MH]^+$, 219.1303. $C_{13}H_{15}FN_2$ requires: $[MH]^+$, 219.1298); ^{1}H NMR (400 MHz, $CDCl_3$) δ 1.37 (9H, s, CH_3), 7.44–7.80 (5H, m, Ar-H); ^{13}C NMR (126 MHz, $CDCl_3$) δ 28.9 (C($\underline{C}H_3)_3$), 31.4 ($\underline{C}(CH_3)_3$), 125.7 (Ar), 128.0 (Ar), 128.7 (Ar), 134.5 (C-3), 141.1 (C-5), 143.9 (d, $^1J_{CF}$ = 247.3 Hz, C-4); ^{19}F NMR (376 MHz, $CDCl_3$) δ -174.7 (s); *m/z* (EI^+) 218.1 ($[M]^+$, 100%), 203.1 (98), 175.0 (32), 163.1 (36), 87.6 (37).

X-ray crystallography

Single crystal X-ray data were collected on Oxford Diffraction Xcalibur Gemini (**4a**) and Bruker SMART 6000 (**4f**) diffractometers equipped with Cryostream (Oxford Cryosystems) nitrogen coolers, at 100 and 120 K, respectively, using graphite-monochromated MoKα radiation (λ = 0.71073 Å). Both structures were solved by direct methods and refined by full-matrix least squares on F^2 for all data using SHELXL [1] and OLEX2 [2] software. All non-disordered non-hydrogen atoms were refined with anisotropic displacement parameters, non-disordered H-atoms in **4a** were located on the difference map and refined isotropically, all H atoms in **4f** were placed in calculated positions and refined in “riding” mode.

Crystallographic data for structures **4a** and **4f** have been deposited with the Cambridge Crystallographic Data Centre as supplementary publications CCDC-829883 and CCDC-829884.

Crystal data for **4a**: $C_5H_7FN_2$, M = 114.13, trigonal, space group R-3c, a = 12.0738(4), c = 20.3220(9) Å, U = 2565.6(2) Å^3, F(000) = 1080, Z = 18, D_c = 1.330 mg m^{-3}, μ = 0.107 mm^{-1}. 8329 reflections yielded 767 unique data (R_{merg} = 0.0471). Final $wR_2(F^2)$ = 0.1266 for all data (50 refined parameters), conventional $R_1(F)$ = 0.0445 for 634 reflections with I>2σ, GOF = 1.047.

Crystal data for **4f**: $C_{11}H_{19}FN_2$, M = 198.28, tetragonal, space group I4$_1$/a, a = 27.1537(9), c = 12.4112(4) Å, U = 9151.1(5) Å^3, F(000) = 3456, Z = 32, D_c = 1.151 mg m^{-3}, μ = 0.080 mm^{-1}. 59470 reflections yielded 5790 unique data (R_{merg} = 0.0782). Final $wR_2(F^2)$ = 0.1994 for all data (266 refined parameters), conventional $R_1(F)$ = 0.0754 for 4844 reflections with I>2σ, GOF = 1.064.

References

1. Sheldrick, G. M. *Acta Cryst.* **2008,** *A64*, 112–122. doi:10.1107/S0108767307043930
2. Dolomanov, O. V.; Bourhis, L. J.; Gildea, R. J.; Howard, J. A. K.; Puschmann, H. *J. Appl. Cryst.* **2009,** *42*, 339–341. doi:10.1107/S0021889808042726
