# Supplementary material for: Continuous gas/liquid–liquid/liquid flow synthesis of 4-fluoropyrazole derivatives by selective direct fluorination
Source: Beilstein J Org Chem. 2011 Aug 2;7:1048–54. doi: 10.3762/bjoc.7.120 (PMC3168861; doi:10.3762/bjoc.7.120)

Supporting Information File 2

for

Continuous gas/liquid–liquid/liquid flow synthesis of 4-fluoropyrazole derivatives by selective direct fluorination

Jessica R. Breen[1], Graham Sandford[1*], Dmitrii S. Yufit[2], Judith A. K. Howard[2], Jonathan Fray[3] and Bhairavi Patel[3]

Address: [1]Department of Chemistry, Durham University, South Road, Durham, DH1 3LE, UK; [2]Chemical Crystallography Group, Department of Chemistry, Durham University, South Road, Durham, DH1 3LE, UK and [3]Pfizer Global Research & Development, Ramsgate Road, Sandwich, Kent, CT13 9NJ, UK

Email: Graham Sandford* - Graham.Sandford@durham.ac.uk

*Corresponding author

NMR spectra

4-Fluoro-3,5-dimethyl-1*H*-pyrazole (4a)

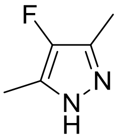

^{1}H NMR (700 MHz, $CDCl_3$, Me_4Si)

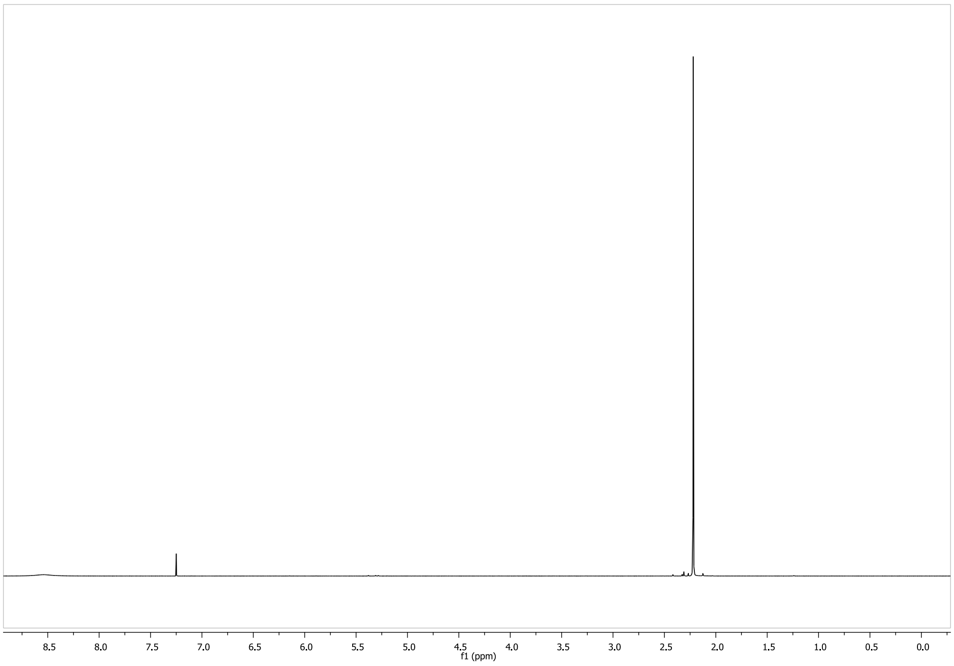

^{19}F NMR (658 MHz, $CDCl_3$)

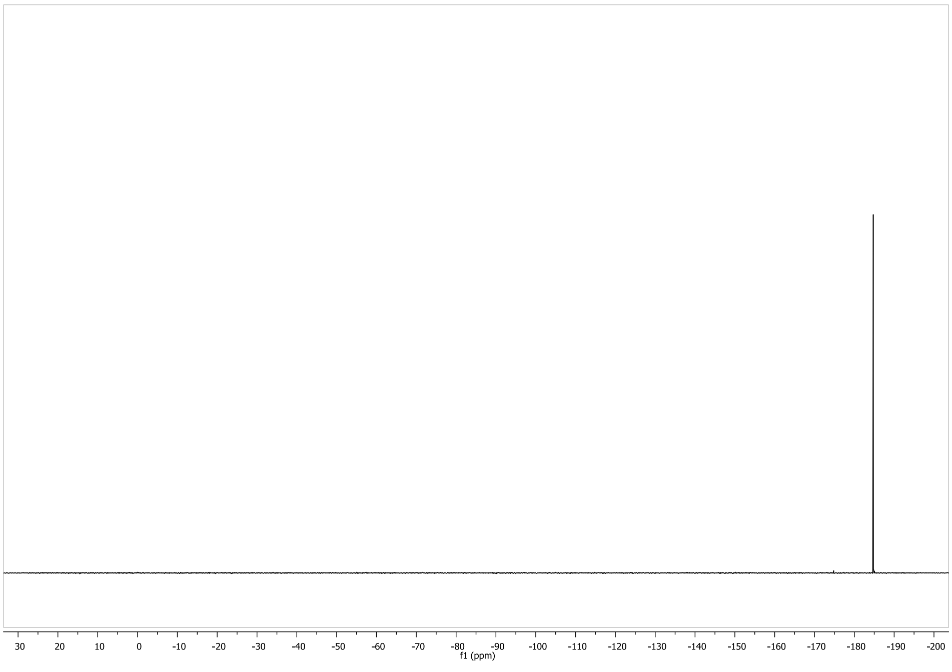

^{13}C NMR (176 MHz, $CDCl_3$, Me_4Si)

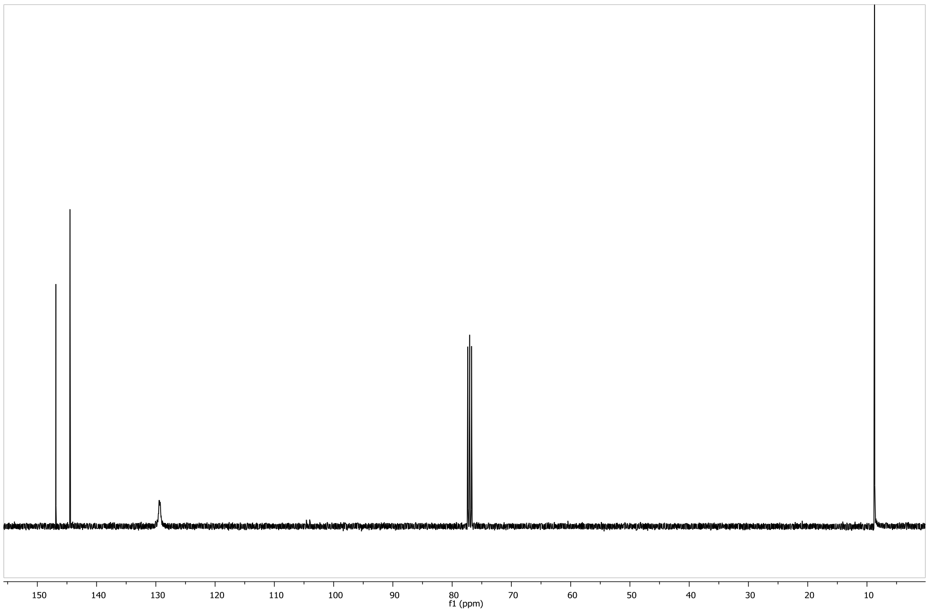

4-Fluoro-1,3,5-trimethyl-1*H*-pyrazole (4b)

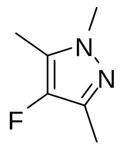

^{1}H NMR (700 MHz, $CDCl_3$, Me_4Si)

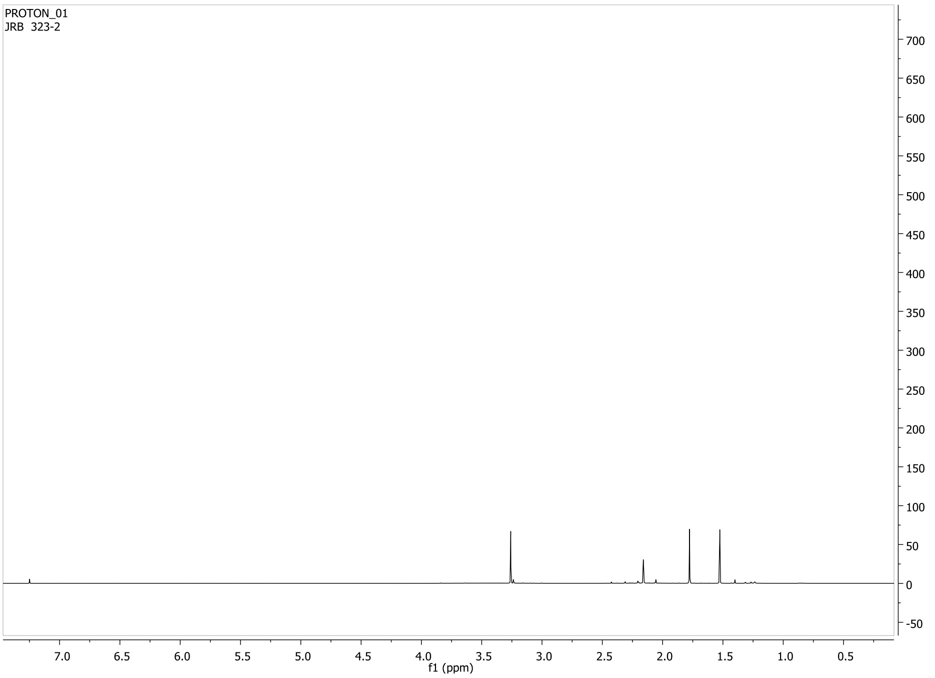

^{19}F NMR (658 MHz, $CDCl_3$, $CFCl_3$)

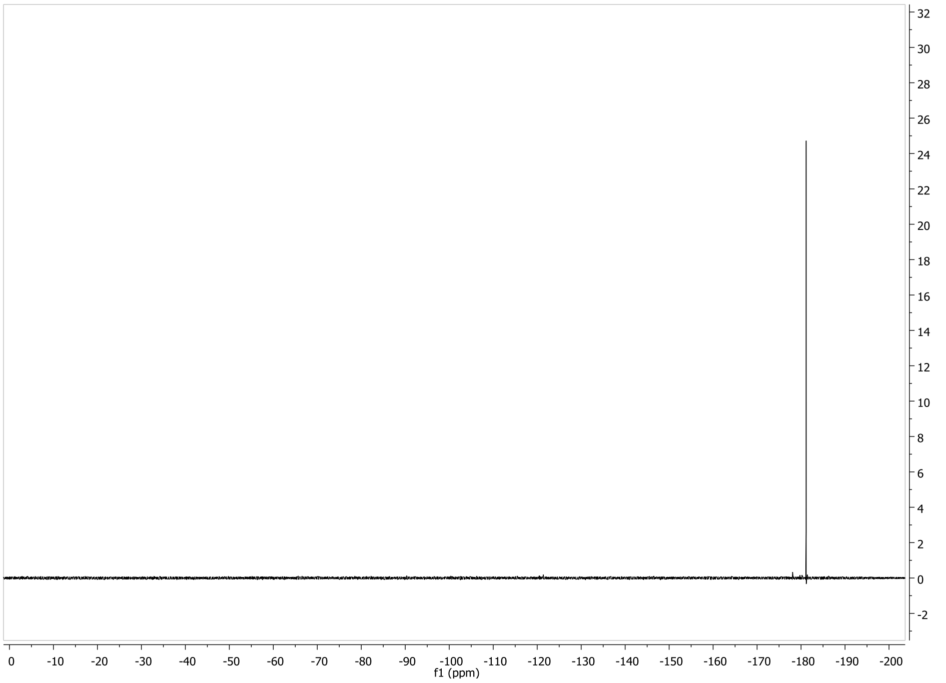

^{13}C NMR (176 MHz, $CDCl_3$, Me_4Si)

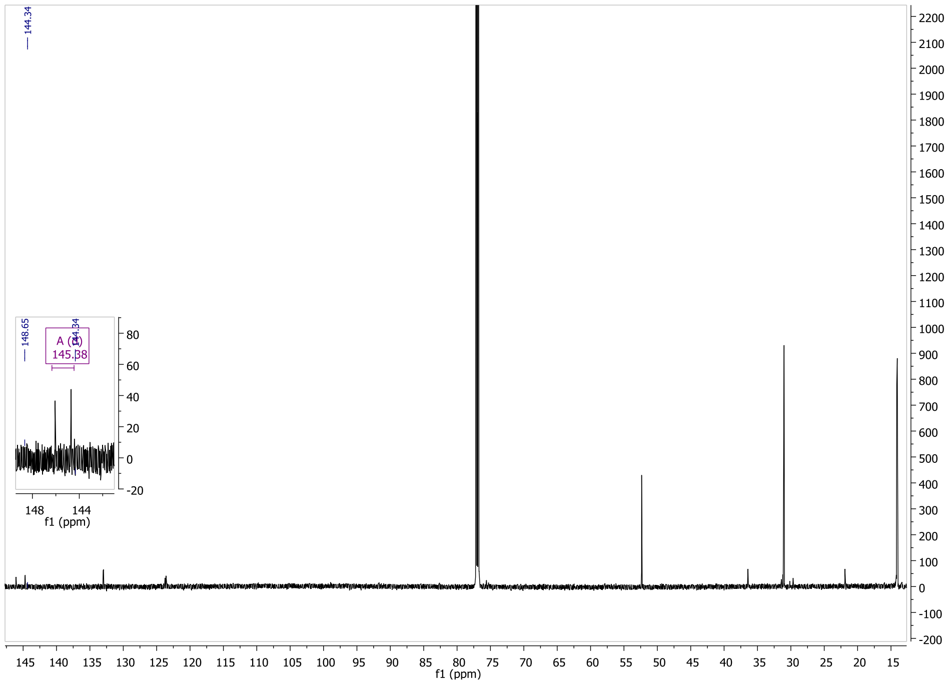

4-Fluoro-3,5-dimethyl-1-phenyl-1*H*-pyrazole (4c)

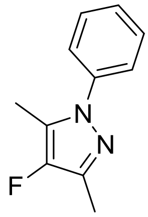

^{1}H NMR (700 MHz, $CDCl_3$, Me_4Si)

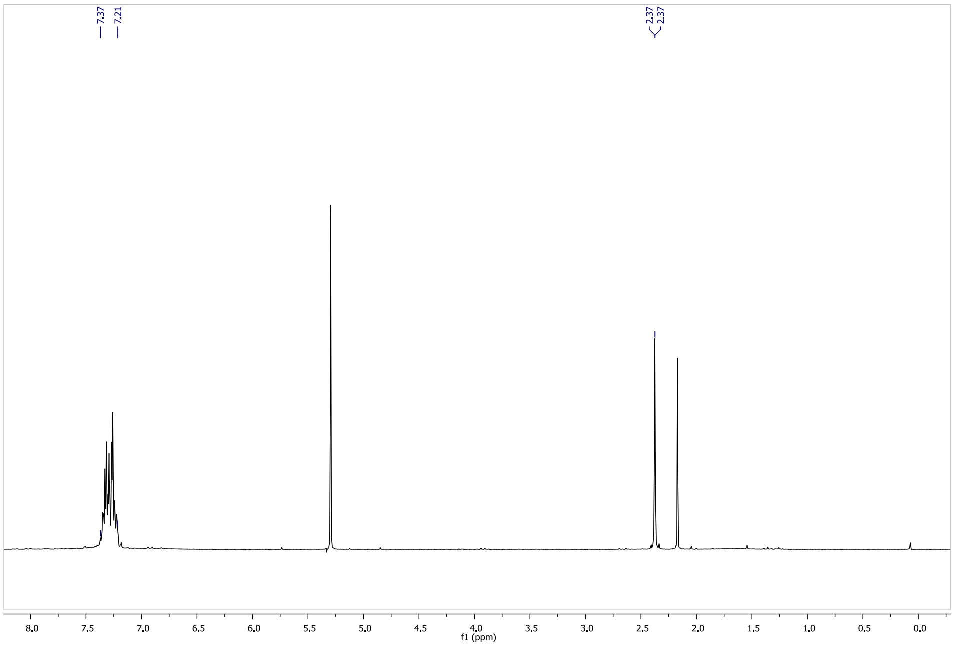

^{19}F NMR (658 MHz, $CDCl_3$, $CFCl_3$)

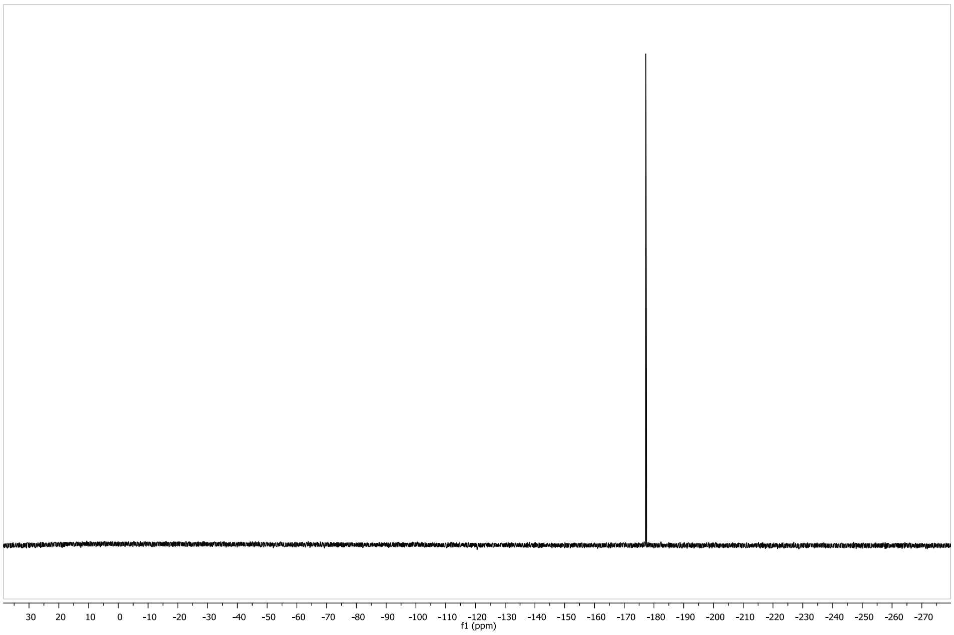

^{13}C NMR (176 MHz, $CDCl_3$, Me_4Si)

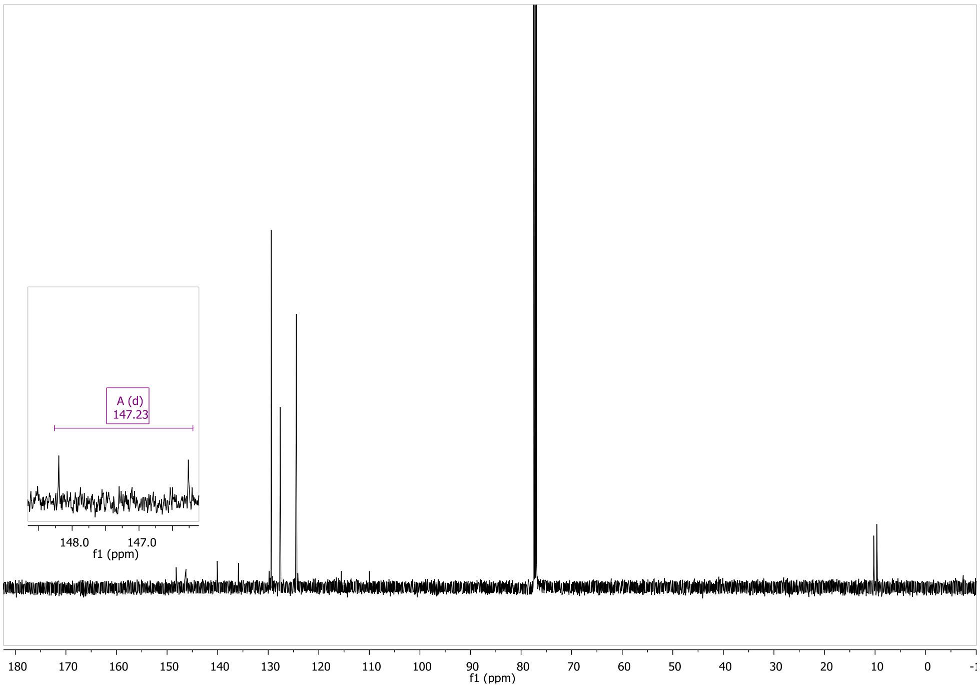

3,5-Diethyl-4-fluoro-1*H*-pyrazole (4d)

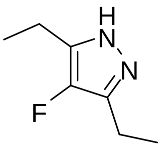

^{1}H NMR (700 MHz, $CDCl_3$, Me_4Si)

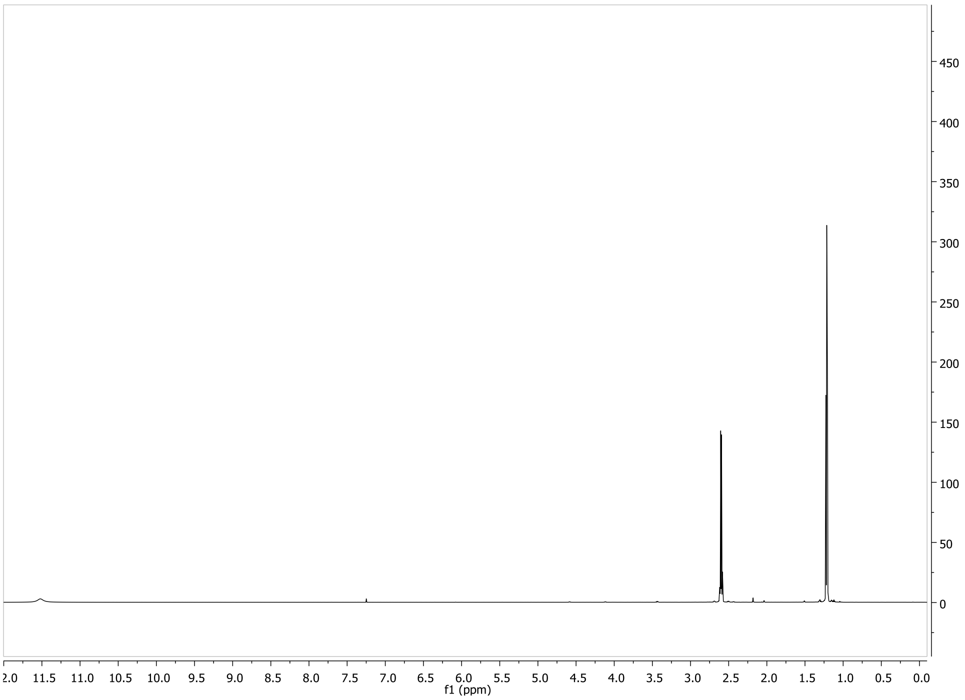

^{19}F NMR (658 MHz, $CDCl_3$, $CFCl_3$)

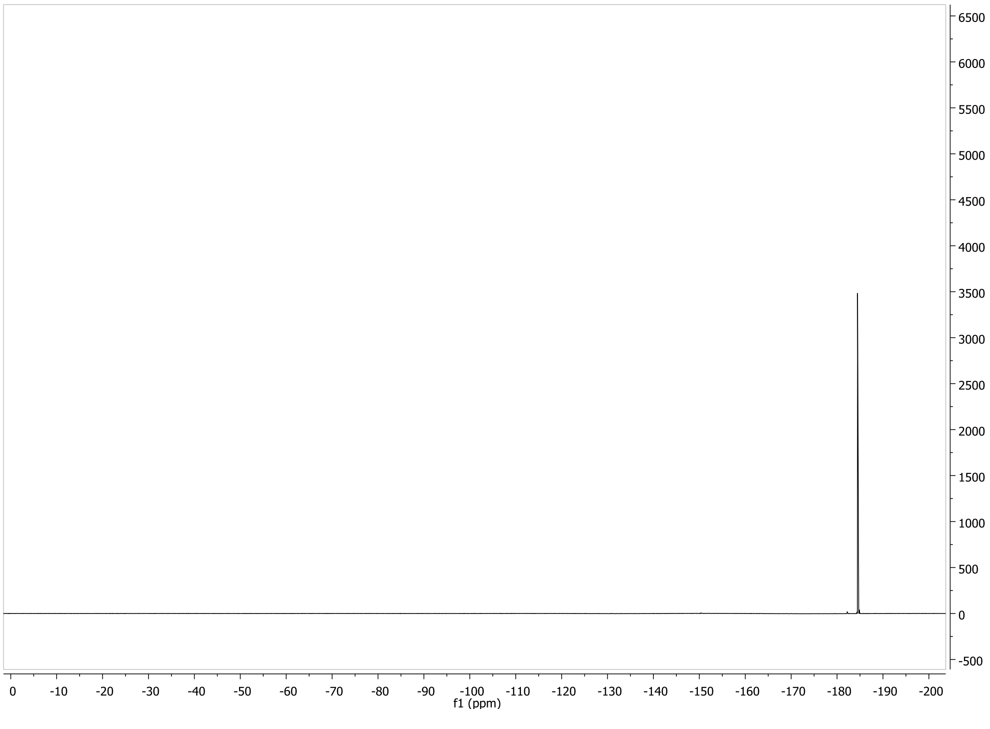

^{13}C NMR (176 MHz, $CDCl_3$, Me_4Si)

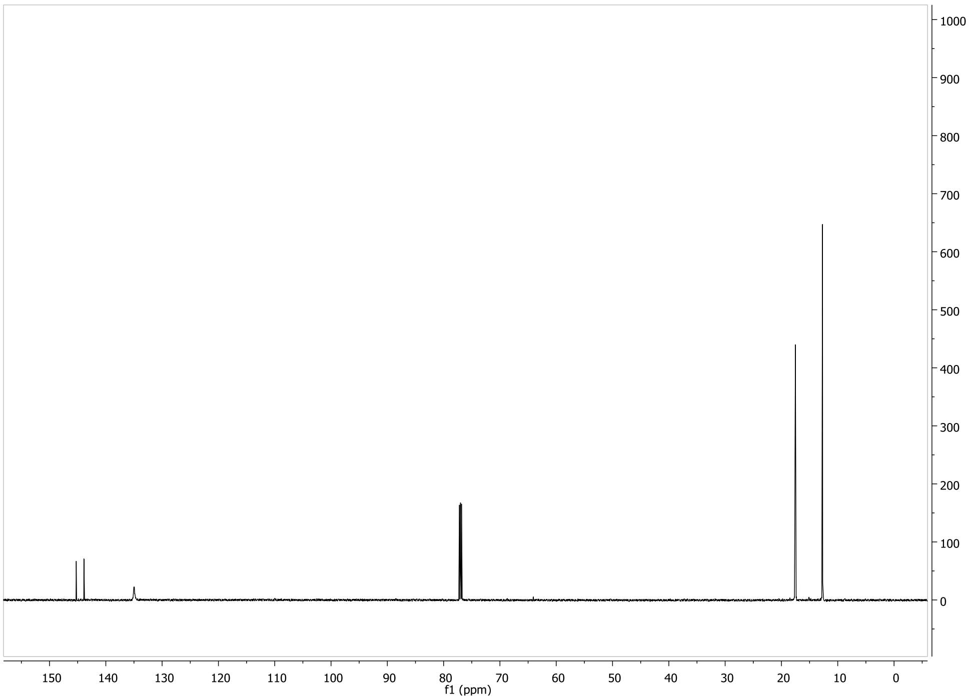

3-*tert*-Butyl-4-fluoro-5-methyl-1*H*-pyrazole (4e)

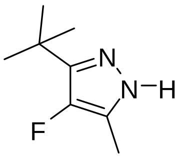

^{1}H NMR (700 MHz, $CDCl_3$, Me_4Si)

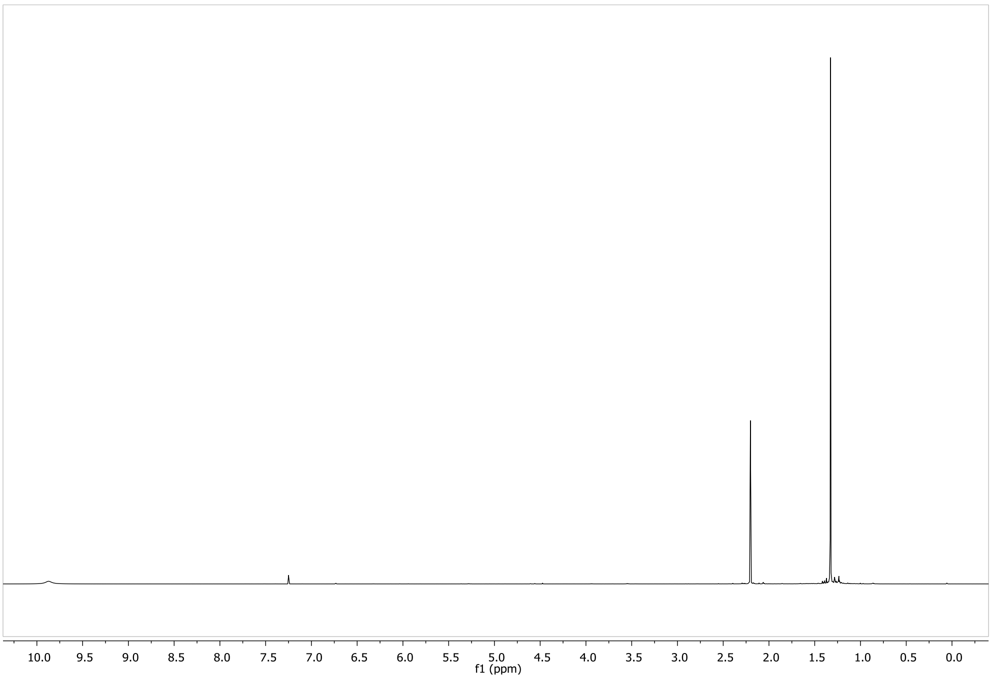

^{19}F NMR (658 MHz, $CDCl_3$, $CFCl_3$)

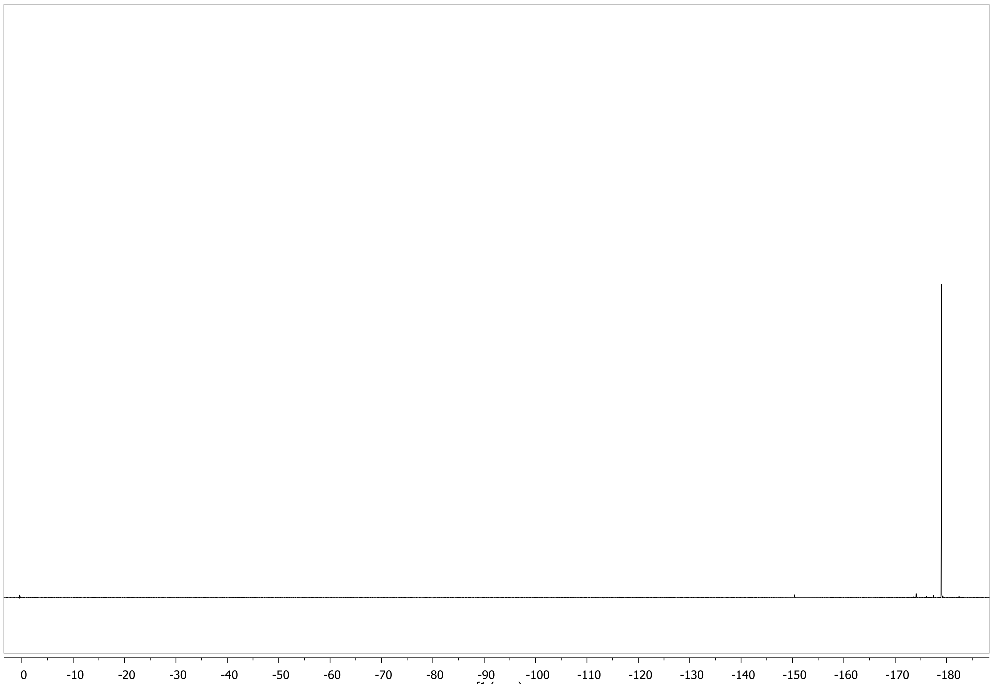

^{13}C NMR (176 MHz, $CDCl_3$, Me_4Si)

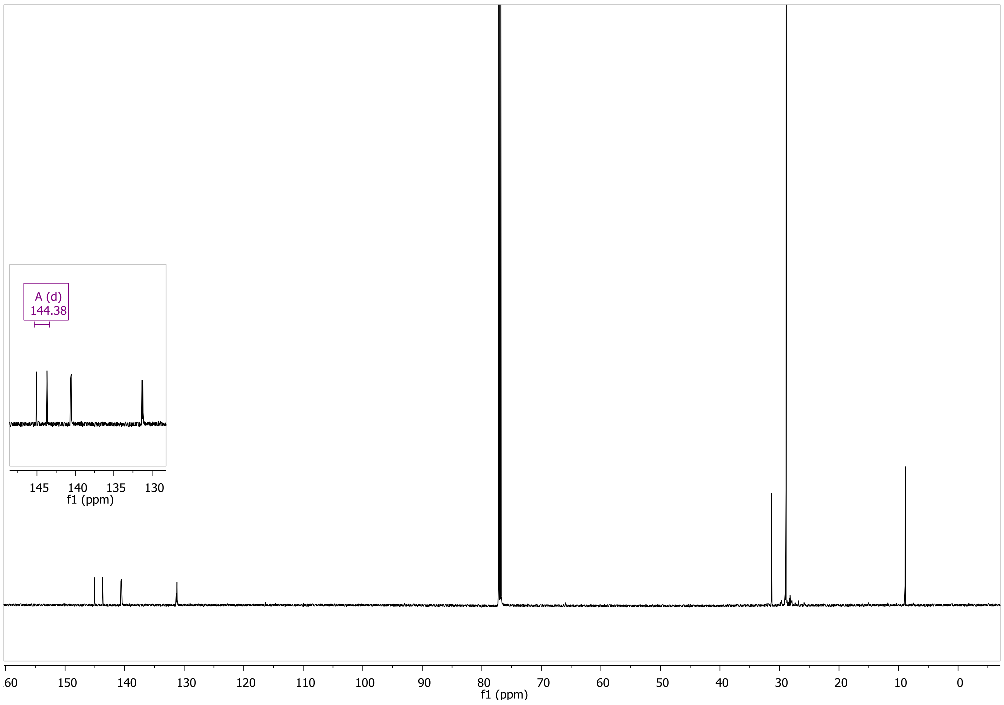

3,5-Di-*tert*-butyl-4-fluoro-1*H*-pyrazole (4f)

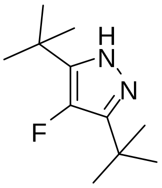

^{1}H NMR (700 MHz, $CDCl_3$, Me_4Si)

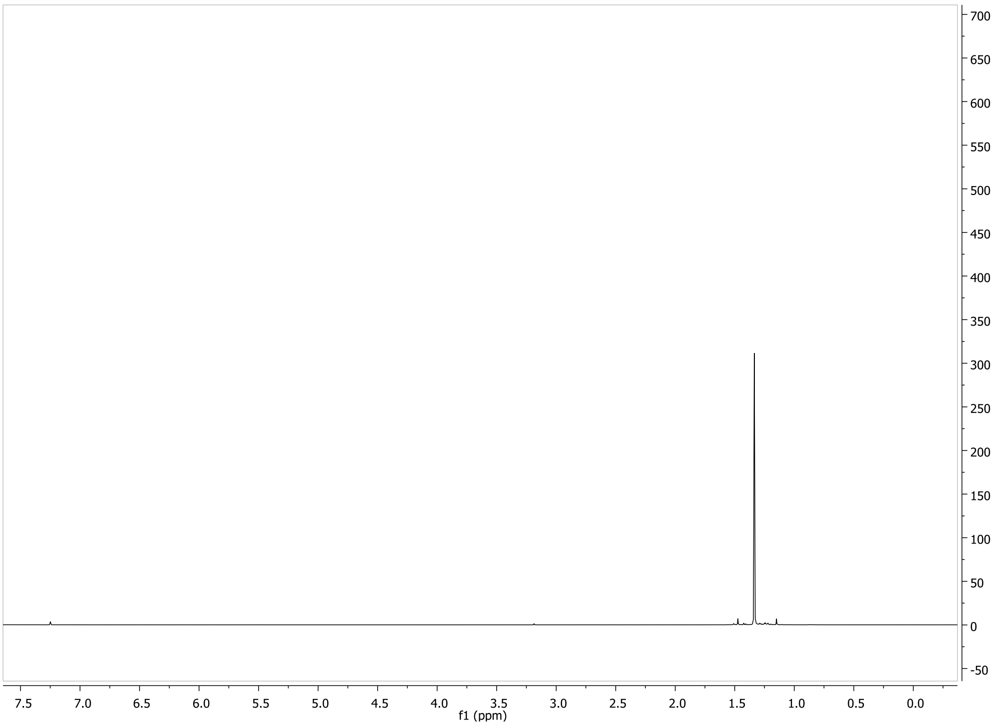

^{19}F NMR (658 MHz, $CDCl_3$, $CFCl_3$)

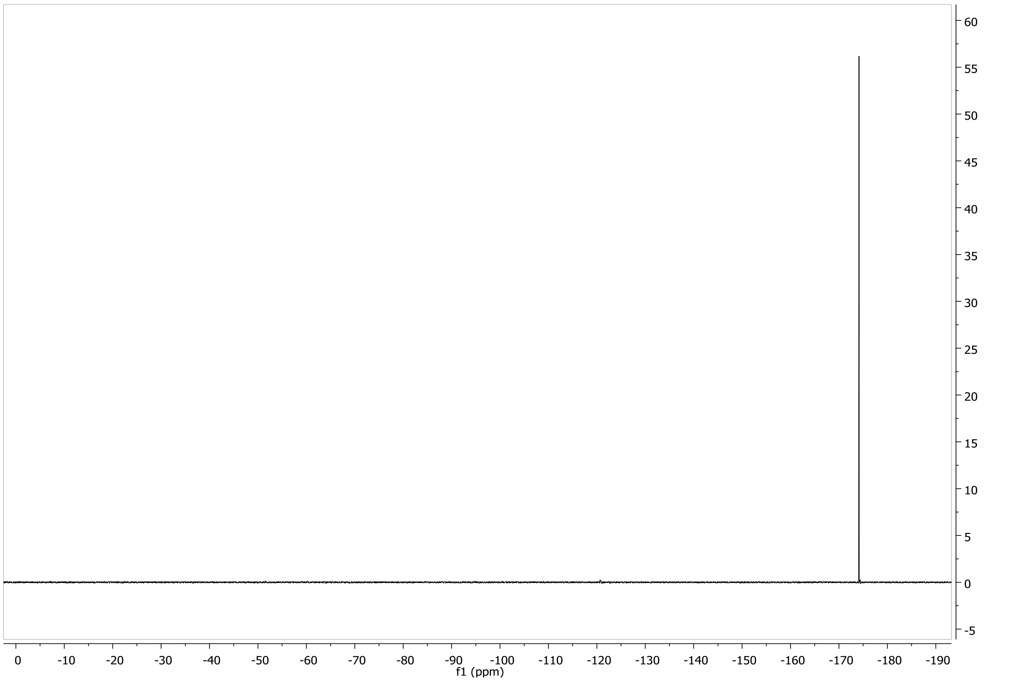

^{13}C NMR (176 MHz, $CDCl_3$, Me_4Si)

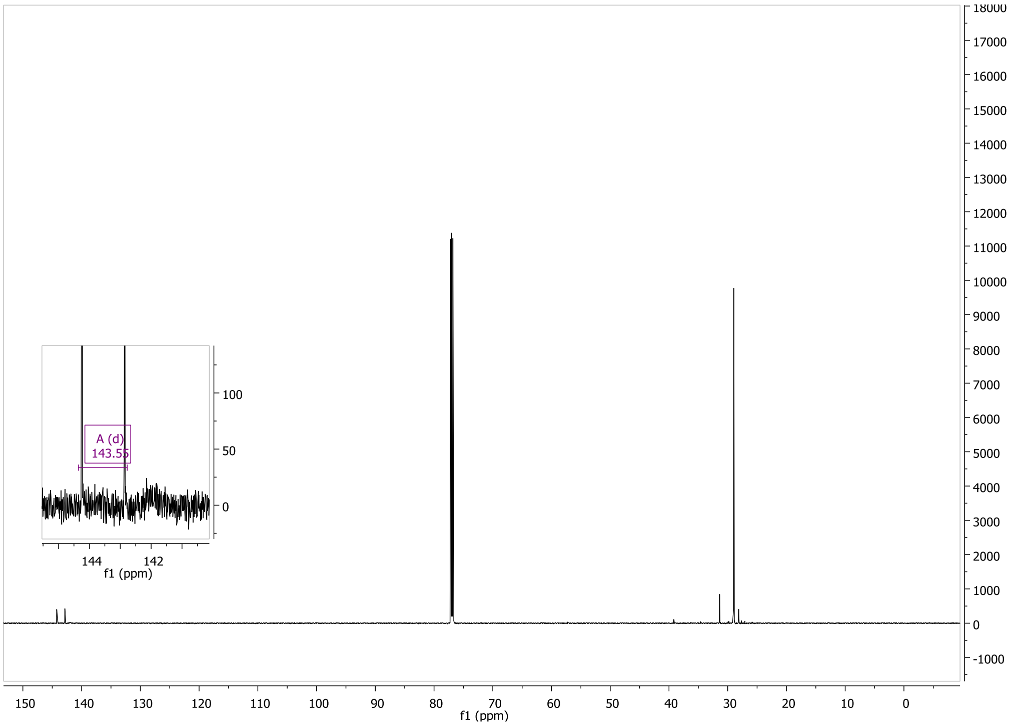

4-Fluoro-5-methyl-3-phenyl-1*H*-pyrazole (4g)

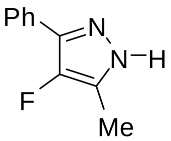

^{1}H NMR (700 MHz, $CDCl_3$, Me_4Si)

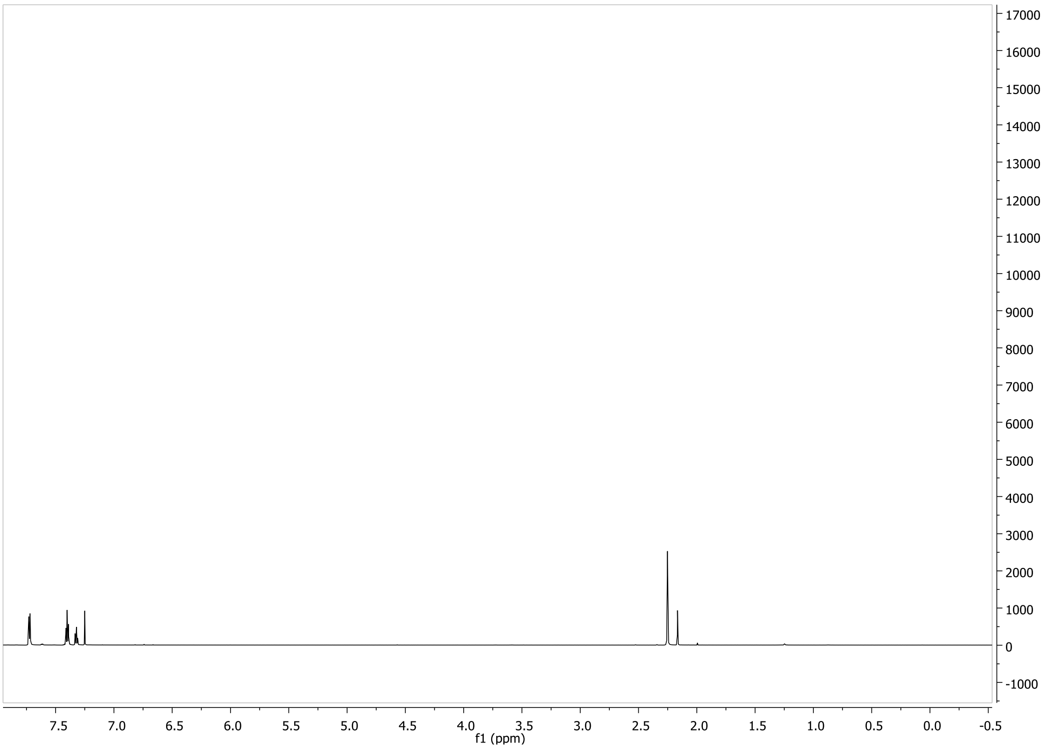

^{19}F NMR (658 MHz, $CDCl_3$, $CFCl_3$)

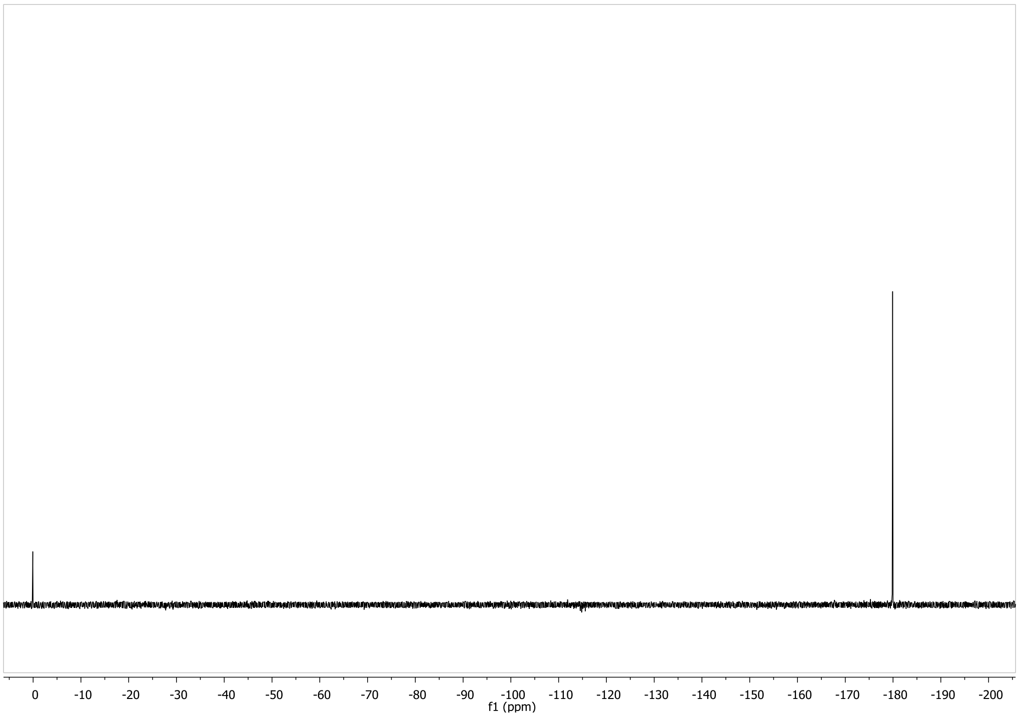

^{13}C NMR (176 MHz, $CDCl_3$, Me_4Si)

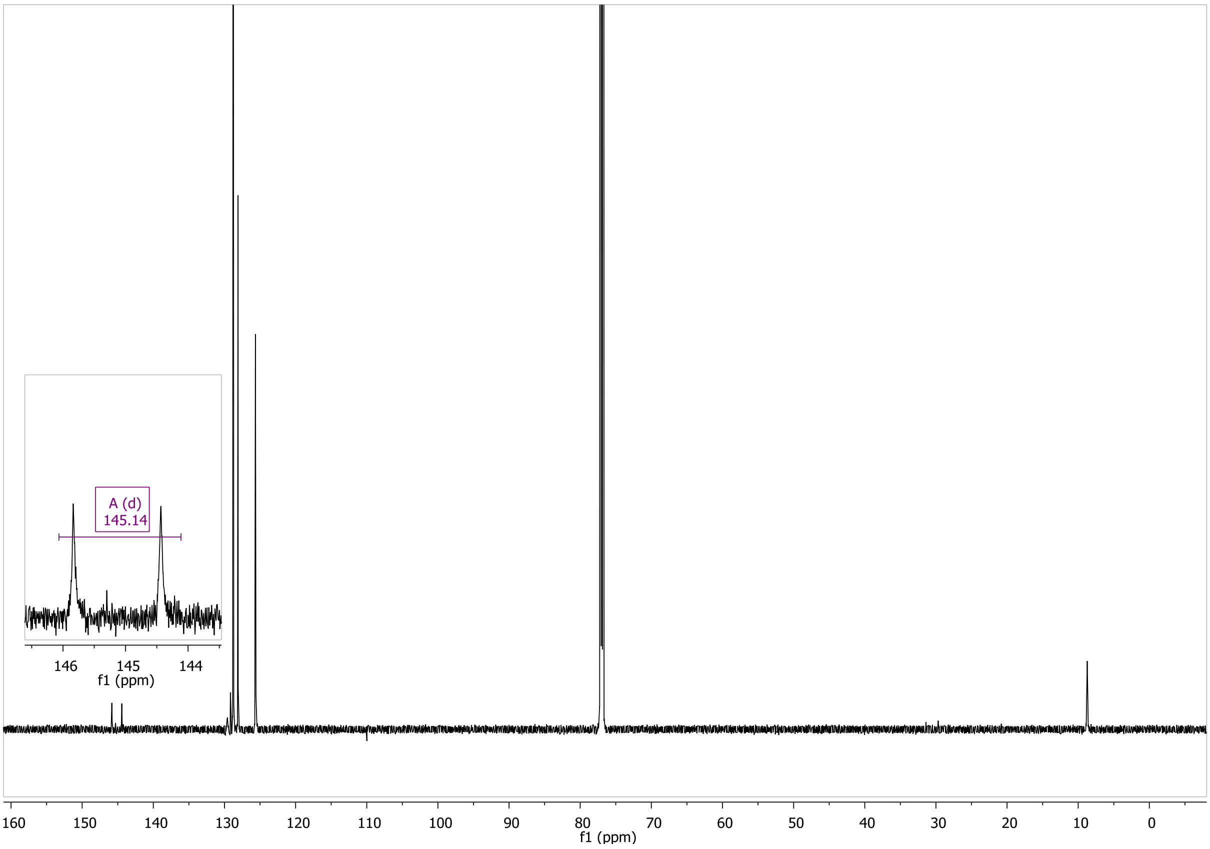

3-*tert*-Butyl-4-fluoro-5-phenyl-1*H*-pyrazole (4h)

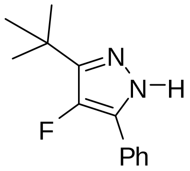

^{1}H NMR (700 MHz, $CDCl_3$, Me_4Si)

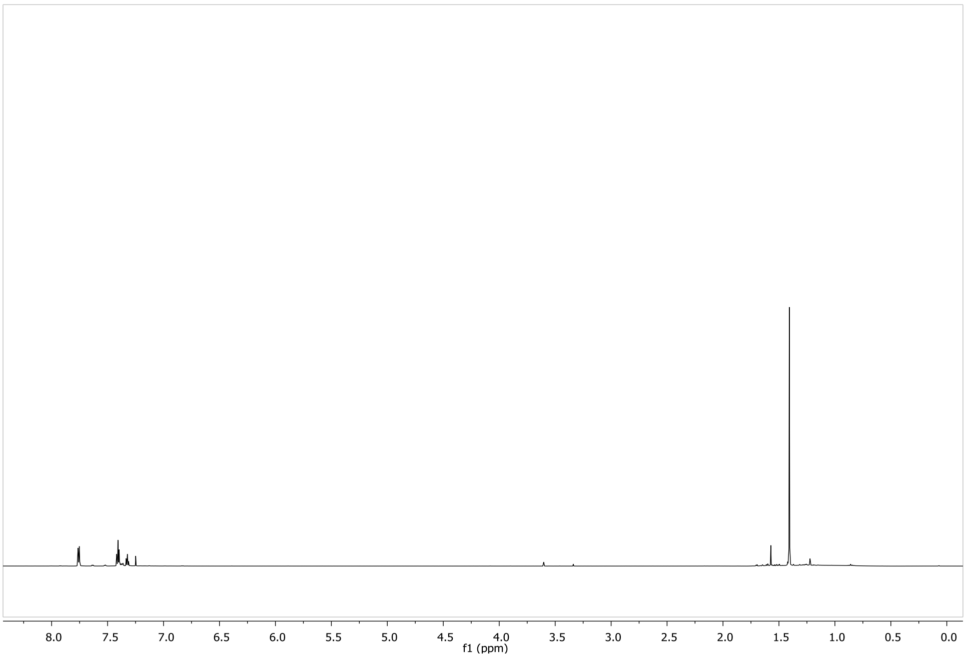

^{19}F NMR (658 MHz, $CDCl_3$, $CFCl_3$)

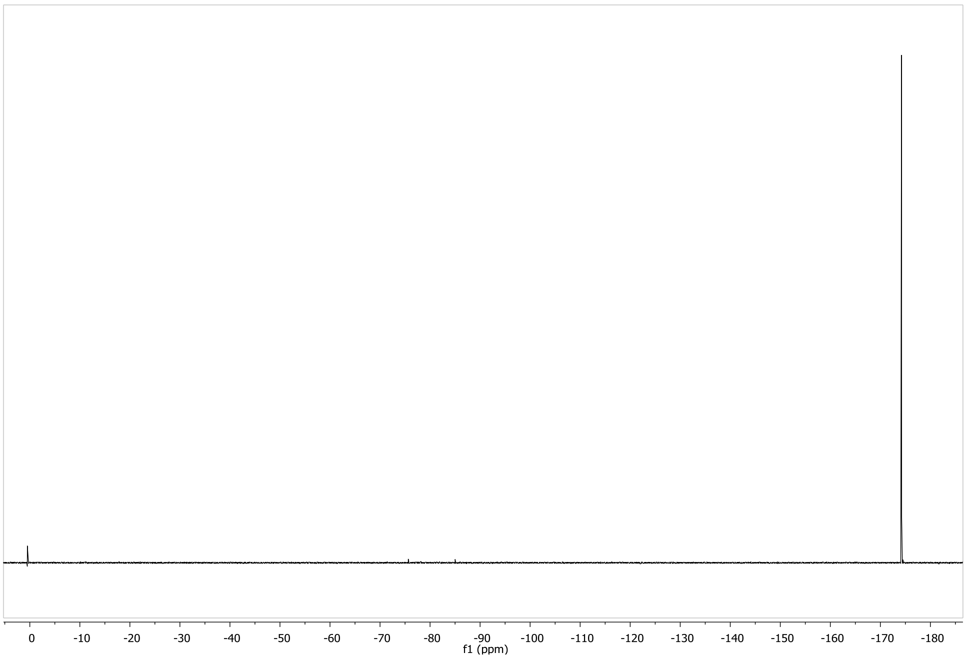

^{13}C NMR (176 MHz, $CDCl_3$, Me_4Si)

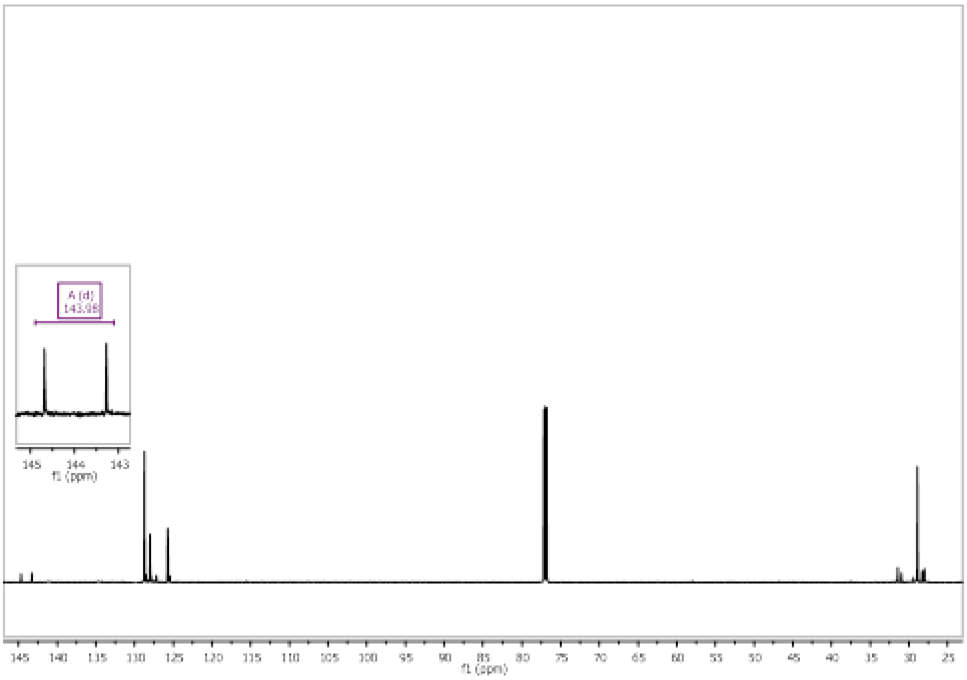

Supplement: File 2 — NMR spectra. [file Beilstein_J_Org_Chem-07-1048-s002.pdf]
